# Prognostic Impact of miR-224 and RAS Mutations in Medullary Thyroid Carcinoma

**DOI:** 10.1155/2017/4915736

**Published:** 2017-06-06

**Authors:** Elisabetta Cavedon, Susi Barollo, Loris Bertazza, Gianmaria Pennelli, Francesca Galuppini, Sara Watutantrige-Fernando, Simona Censi, Maurizio Iacobone, Clara Benna, Federica Vianello, Stefania Zovato, Davide Nacamulli, Caterina Mian

**Affiliations:** ^1^Familial Tumor Unit, Veneto Institute of Oncology, (IOV)-IRCCS, Padova, Italy; ^2^Endocrinology Unit, Department of Medicine (DIMED), University of Padua, Padua, Italy; ^3^Surgical Pathology & Cytopathology Unit, Department of Medicine (DIMED), University of Padua, Padua, Italy; ^4^Surgery Unit, Department of Surgical, Oncological and Gastroenterological Sciences (DISCOG), University of Padua, Padua, Italy; ^5^Department of Radiotherapy, Veneto Institute of Oncology (IOV)-IRCCS, Padova, Italy

## Abstract

Little is known about the function of microRNA-224 (miR-224) in medullary thyroid cancer (MTC). This study investigated the role of miR-224 expression in MTC and correlated it with mutation status in sporadic MTCs. A consecutive series of 134 MTCs were considered. Patients had a sporadic form in 80% of cases (107/134). In this group, REarranged during transfection (*RET*) and rat sarcoma (*RAS*) mutation status were assessed by direct sequencing in the tumor tissues. Quantitative real-time polymerase chain reaction was used to quantify mature hsa-miR-224 in tumor tissue. *RAS* (10/107 cases, 9%) and *RET* (39/107 cases, 36%) mutations were mutually exclusive in sporadic cases. miR-224 expression was significantly downregulated in patients with the following: high calcitonin levels at diagnosis (*p* = 0.03, *r* = −0.3); advanced stage (*p* = 0.001); persistent disease (*p* = 0.001); progressive disease (*p* = 0.002); and disease-related death (*p* = 0.0001). We found a significant positive correlation between miR-224 expression and somatic *RAS* mutations (*p* = 0.007). Patients whose MTCs had a low miR-224 expression tended to have a shorter overall survival (log-rank test *p* = 0.005). On multivariate analysis, miR-224 represented an independent prognostic marker. Our data indicate that miR-224 is upregulated in *RAS*-mutated MTCs and in patients with a better prognosis and could represent an independent prognostic marker in MTC patients.

## 1. Introduction

Medullary thyroid carcinoma (MTC) is a rare neuroendocrine cancer originating from parafollicular, calcitonin- (Ct-) producing C-cells. It accounts for 5–10% of all thyroid carcinomas with a global 10-year survival rate around 65% to 70%. About 75% of MTCs are sporadic, while the remainders are hereditary, due to germline mutations that activate the REarranged during transfection (*RET*) proto-oncogene [1]. Various clinical, pathological, and genetic variables have been proposed as prognostic markers, including serum calcitonin (Ct) levels at diagnosis, extent of nodal disease, distant metastasis, pathological stage, and mutational damage in tumor suppressor genes [2]. Distinctive germinal *RET* mutations in the inherited forms and somatic *RET* mutations in sporadic cases represent the most important molecular markers for an adequate prognostic stratification of MTC patients [3–6].

It has been demonstrated that a combined analysis of somatic *RET* and Ki-67 is useful for identifying patients with a more aggressive cancer, and their joint assessment could ameliorate the initial risk stratification of patients with sporadic MTC, and thus be of prognostic relevance [7].

Moura et al. recently reported finding *RAS* somatic mutations in 68% of *RET*-negative sporadic MTCs. *RAS* mutations were detected only in *H-RAS* and *K-RAS* genes (not in *N-RAS* genes), apparently representing an alternative genetic event to *RET* mutations in sporadic MTC. These results were reproduced by others, who found proportion *RAS*-mutated cases ranging from 0% to 26.2% of MTCs. According to a recent analysis conducted by Ciampi et al. on one of the largest series in the literature [8], the average prevalence of *RAS* mutations in sporadic MTC is around 8.8%, and these authors confirmed that *RET* and *RAS* mutations were mutually exclusive. On the other hand, 40–60% of sporadic MTCs remained unassociated with any recognized genetic event.

The discovery of single strands of noncoding RNA in the human genome and their role in modulating gene expression at post-transcriptional level represent a great breakthrough in the postgenome sequencing era. MicroRNAs are small and bind to the 3′-untranslated region of target genes, suppressing translation and/or causing mRNA degradation. That is why microRNA can play an important part in essential processes such as cell differentiation, growth, and cell death [9]. Investigating the role of microRNAs is an essential aspect of cancer research [10], and there has been a growing interest of late in how they may influence the pathogenesis and prognosis of MTC [11–15]. In particular, the role of miR-224 in cancer is under investigation and has yet to be clearly established. It seems to be a negative prognostic factor in lung adenocarcinoma, colorectal cancers, hepatocellular carcinoma, and cervical carcinoma [16–19]. On the other hand, several recent studies have identified its overexpression as a marker of a greater radio-sensitivity in glioblastoma and chemo-sensibility in prostatic carcinoma [20, 21]. These findings indicate that miR-224 has an indispensable role in cell proliferation, but also in the apoptosis of cancer cells, and the crucial balance between these two processes decides the miR-224 phenotype identifiable in tumor cells [22].

The aims of the present study on a large series of familial and sporadic cases of MTC were as follows: (a) to confirm our previous findings concerning miR-224 expression and its relationship with patient outcome; (b) to elucidate its relationship with the main molecular events responsible for MTC.

## 2. Materials and Methods

### 2.1. Patients

The cases considered were retrospectively selected from the electronic archives of the Surgical Pathology and Cytopathology Unit at the University of Padua, based on the elevated calcitonin levels and the diagnosis on MTC. All patients involved in this study gave their written informed consent, and the institute's ethical regulations on research on human tissues were followed.

The study concerned a consecutive series of 134 patients with MTC (107 sporadic and 27 familial; 54 males and 80 females; median age 59, range 5–87 years) collected from 2006 to 2015 with a median follow-up of 40 months (range 1–140 months). Serum Ct levels at diagnosis were available for most all the patients, who were considered biochemically cured if they had basal Ct levels below 10 pg/ml a year after primary surgery, and/or at the latest follow-up. Disease progression status was defined based on increasing disease burden, according to RECIST criteria, and/or on Ct/CEA-doubling times lower than 24 months. Patients were considered with stable disease if Ct/CEA-doubling times were higher than 24 months, without increasing disease burden [4].

At the end of the study period, 65% of patients (86/133) were biochemically disease-free judging from their latest Ct test (which was unavailable for 1 patient). During the study period, 13% of the patients (17/134) had progressive disease, and 8 of them were treated with tyrosine kinase inhibitors (TKI). By the end of the study period, 7% (9/132) had died of their disease.

### 2.2. RET and RAS Analysis

For each cancer sample, before DNA/RNA extraction, two experienced pathologists (F. G. and G. P.) analyzed a frozen section slightly colored by hematoxylin and eosin confirming MTC diagnosis and checking that at least 70% of cancer cells were present. DNA was extracted from tissue and from whole-blood samples using the DNeasy Blood and Tissue kit (Qiagen, Milano, Italy) according to the manufacturer's protocol in order to establish their mutational status and whether any mutations were germline or sporadic. Analyses were run for *RET* (NM_020975.4) (exons 5, 8, 10, 11, 13, 14, 15, and 16), *N-RAS* (NM_002524.3) (exons 2 and 3), *K-RAS* (NM_033360.2) (exons 2 and 3), and *H-RAS* (NM_005343.2) (exons 2 and 3) mutations by direct sequencing (bidirectionally, as standard practice in positive samples), as explained elsewhere [7, 23]. When *RET* or *RAS* mutations were found, we confirmed the results by a new amplification PCR product and then bidirectionally sequencing.

### 2.3. miR-224 Quantitative Real-Time Polymerase Chain Reaction

Tissue cores were deparaffinized with xylene at 50°C for 3 min. Total RNA extraction was done using the RecoverAll kit (Ambion, Austin, Texas, USA) according to the manufacturer's instructions. The NCode™ miRNA qRT-PCR method (Invitrogen, Carlsbad, California, USA) was used to detect and quantify mature hsa-miR-224 (primer: 5′-gca agt cac tag tgg ttc cgt t-3′) on a real-time LightCycler 480 instrument, according to the manufacturer's instructions (Roche, Milan, Italy). Normalization was performed with the small nuclear RNA U6B (RNU6B; primer: 5′-acg caaattcgtgaagcg tt-3′). Data were analyzed using the comparative cycle threshold (CT) method by LightCycler® Relative Quantification Software, 2001. All real-time reactions, including no template controls, were run in duplicate, as described elsewhere [24].

### 2.4. Statistical Analysis

All statistical analyses were performed using the *MedCalc* software (rel. 11.6.0). The Kolmogorov-Smirnov test was used to assess the normal distribution of each variable. After logarithmic transformation, the *t*-test and ANOVA were used to measure differences in miR-224 expression levels in different subgroups based on clinical/pathological variables (female versus male, different stages at diagnosis, lower versus higher Ct levels at diagnosis, biochemically cured status versus persistent disease, disease progression versus a stable disease, and death versus alive) and molecular features (*RET*/*RAS* status). The Mann-Whitney test and Kruskal-Wallis test for nonparametric data were used to correlate Ct levels with pathological and molecular data and patient outcome. Fisher's exact test was used to study the influence of somatic mutations on patient outcome in sporadic MTC cases. A rank correlation analysis was used to study the influence of miR-224 on CT levels at diagnosis. The Kaplan-Meier method was used to estimate the survival rates, and the log-rank test was used to assess the survival differences between the groups (the miR-224 levels were dichotomized as “high” or “low” based on the mean value of the variable). Multiple logistic regression was also used to ascertain the independent effect of miR-224 and the clinical and pathological features considered on the outcome of MTC patients. Differences were considered statistically significant when *p* was less than 0.05.

## 3. Results

Table 1 shows patients' clinical and pathological findings and their molecular *RET*/*RAS* status.

We initially examined the clinical/pathological differences in relation to serum Ct levels at diagnosis, then we considered the differences in miR-224 expression level in relation to the following: (a) clinical/pathological findings (CT level at diagnosis, nodal and distant involvement, and TNM stage); (b) patient's outcome; and (c) somatic *RET* and *RAS* mutations in sporadic MTCs.

As expected, higher serum Ct levels at diagnosis correlated positively with the presence of nodal and distant metastases, higher stage disease at diagnosis (for all variables, *p* = 0.0001; *t*-test), persistent disease during the follow-up (*p* = 0.0001; *t*-test), and a higher risk of disease-related death (*p* = 0.0001; *t*-test).

**Figure 1:**
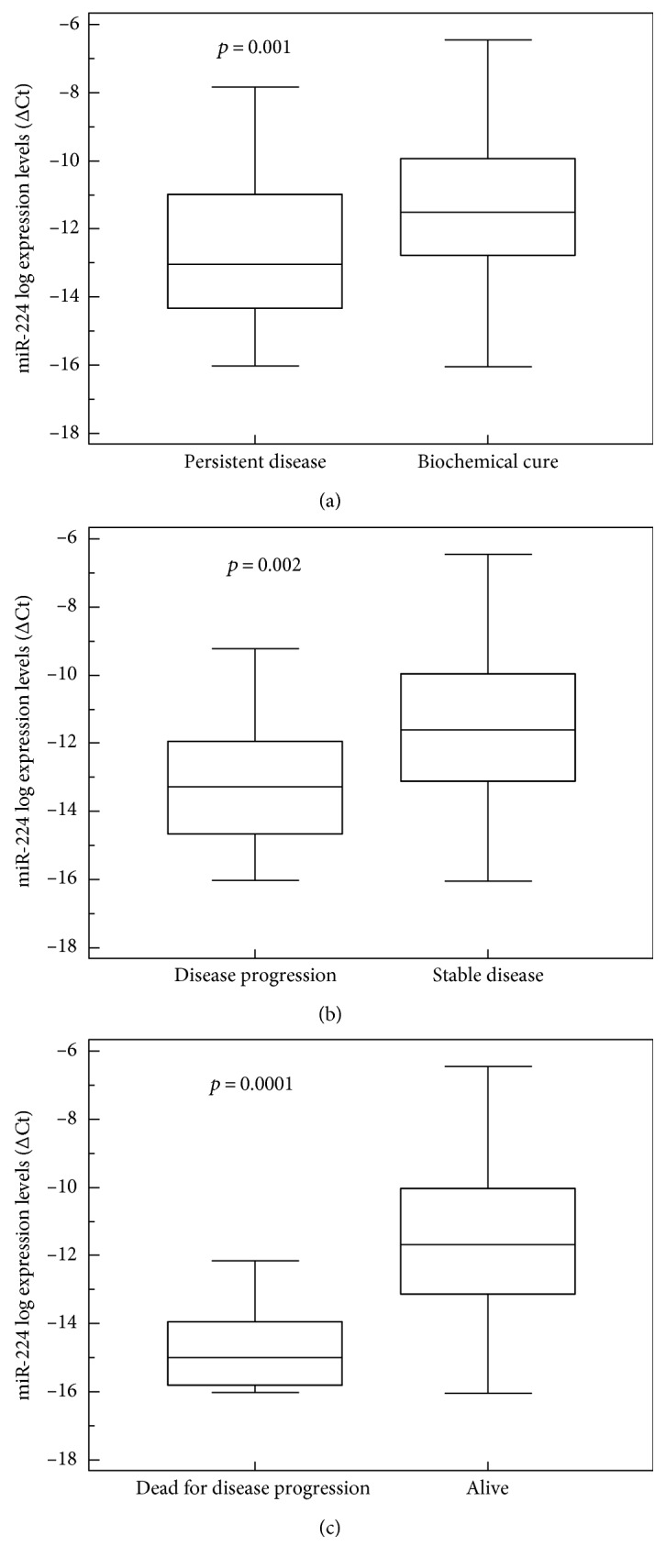
(a) Correlation between miR-224 expression level and persistent disease: lower levels of miR-224 were associated with higher risk of persistent (box-and-whisker plot, *p* = 0.001). (b) Correlation between miR-224 expression level and disease progression: lower levels of miR-224 were associated with higher risk of disease progression (box-and-whisker plot, *p* = 0.002). (c) miR-224 expression levels and death for disease progression. At the end of the follow-up, patients who died with progressive disease had lower miR-224 levels (box-and-whisker plot, *p* = 0.0001).

**Figure 2:**
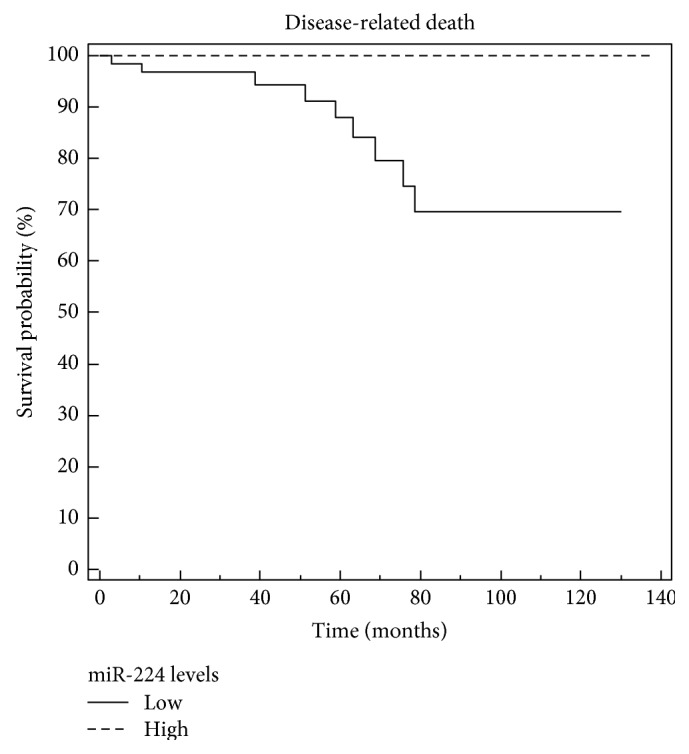
miR-224 and survival curve. On Kaplan-Meier analysis, patients with lower miR-224 expression levels tended to have a shorter survival than those with higher miR-224 levels. The miR-224 levels were dichotomized as “high” or “low” based on the mean value of the variable (log-rank test *p* = 0.005).

The expression levels of miR-224 were significantly associated with patients' main clinical and pathological findings and with their outcome: there was a statistically significant negative association between miR-224 expression level and serum Ct level at diagnosis: the higher the former, the lower the latter (*p* = 0.03, *r* = −0.3; rank correlation). We found lower miR-224 values in patients with advanced stage disease at diagnosis (*p* = 0.001; ANOVA): patients with nodal and distant metastases at diagnosis had significantly lower miR-224 levels (*p* = 0.005 and *p* = 0.0001, resp.; *t*-test). We also found significantly lower miR-224 expression levels during the study period in patients with persistent disease, progressive disease, and fatal disease progression (*p* = 0.001, *p* = 0.002, and *p* = 0.0001, resp.; *t*-test) (Figure 1). As expected from the Kaplan-Meier analysis and log-rank test, patients with a low miR-224 expression tended to have a shorter overall survival than those with a high miR-224 expression levels (*p* = 0.005; log-rank test) (Figure 2).

We found somatic *RET* mutations in 36% of cases of sporadic MTC (39/107), most of them located on exon 16 at codon 918 (M918T in 23/39; 59%). In one patient (2.6%), RET mutation was on exon 8 (A513G), in 2 (5.1%) on exon 10 (C609S, C618R), and in 8 (20.5%) on exon 11 (2 with a C630R, 1 with a C630S, 2 with a C634Y, 1 with a C634W, 1 with a del613E, and 1 patient with a p.L629_L633del.). Five patients (12.8%) had their RET mutation on exon 15 (3 with a A883F and 2 with a p.D898_E901del). Somatic *RAS* mutations were found in 10 sporadic MTC patients (10/107; 9%): 3 had a mutation in *K-RAS* (two G12R and one G13R); one had a mutation in *N-RAS* (Q61L); and 6 had a mutation in *H-RAS* (two Q61R, one Q61K, one M72I, one G12R, and one was a novel G60D mutation, never previously reported in the literature). This last novel mutation warrants further study to clarify its role and importance, and the patient carrying this G60D mutation was consequently excluded from the statistical analysis. Here again, we confirmed that *RAS* and *RET* sporadic mutations were mutually exclusive.

In the sporadic cases, *RET* somatic mutations correlated with the following: the presence of nodal metastases (*p* = 0.02, Fisher's exact test); distant metastases at diagnosis (*p* = 0.01, Fischer's exact test); and advanced stage at diagnosis (*p* = 0.02; Fisher's exact test). *RET* mutations also correlated with persistent disease at the end of the follow-up and an increased risk of disease-related death: the prevalence of *RET* mutations was significantly lower among patients who were cured than among those with persistent disease at the latest follow-up (28% versus 50%, *p* = 0.04; Fisher's exact test), while it was much higher among patients who died of their disease than among those still alive (78% versus 32%, *p* = 0.02; Fisher's exact test). We found no significant association between the presence of *RET* mutations and miR-224 expression levels. Furthermore, when we compared MTC outcome according to different *RET* mutations (M918T cases versus non-M918T ones), we found a significant association between the presence of the M918T mutation and persistent disease (*p* = 0.02, by chi-squared test). No other significant association was found.

**Figure 3:**
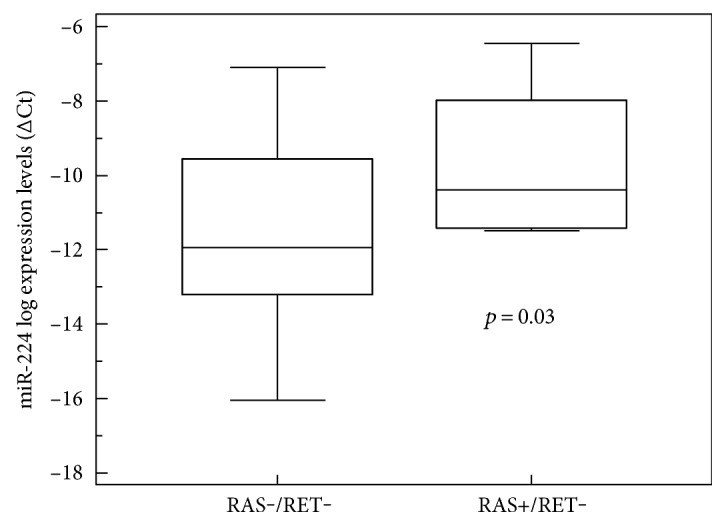
Correlation between miR-224 expression level and mutational status. The presence of a RAS mutation was associated with higher levels of miR-224 (box-and-whisker plot, *p* = 0.03).

When we considered the associations between the presence of *RAS* somatic mutations and the main clinical and pathological variables, we found no statistically significant correlations. However, all *RAS*-mutated patients were alive at the end of the study period: one had biochemical evidence of disease with stably low serum Ct levels (around 45 ng/L); the others were biochemically cured. We also found a significant positive association between *RAS-*mutated status and miR-224 expression levels: the presence of *RAS* mutations was associated with higher miR-224 levels (*p* = 0.007; *t*-test), and the association was confirmed after the exclusion of RET-positive patients, too (*p* = 0.03; *t*-test), confirming that sporadic *RAS*+/RET−MTC is a less aggressive phenotype (Figure 3). In addition, in Table 2, we reported clinical data based on *RET+/RAS−* versus *RET−/RAS−* versus *RET−/RAS+* to clearly highlight the different outcomes due to the mutational profile.

Finally, our multivariate logistic regression analysis demonstrated that lower serum Ct levels at diagnosis and higher levels of miR-224 expression correlated independently with biochemical cure (OR 0.999, 95%CI 0.9987–0.999 and OR 1.4, 95%CI 1.06–1.8, resp.). Stage at diagnosis and miR-224 expression levels correlated independently with disease progression (OR 29.8, 95%CI 3.7–243.3 and OR 0.7, 95%CI 0.5–0.9, resp.), but only miR-224 levels correlated independently with disease-related death (OR 0.3, 95%CI 0.1–0.6).

## 4. Discussion

It has been estimated that more than 500 miRNAs are expressed in humans [25]. These miRNAs are believed to regulate the expression of nearly 5000 human genes or 30% of all human proteins. Recent evidence has shown that the interactions between miRNAs and their numerous mRNA targets may have important roles in gene control inside the cells and may be implicated in a variety of biological processes, such as cellular differentiation, proliferation, and apoptosis. By fine-adjusting gene expression, miRNAs may govern aspects that are crucial in determining cancer phenotypes (i.e., signaling, differentiation, invasion, and metastasis). They target both tumor suppressor and oncogenic pathways [26], and their altered expression carries great diagnostic and prognostic potential [27–29]. In addition, miRNAs are highly stable and can be detected reliably in archival clinical samples and cytology specimens and are therefore considered ideal candidate biomarkers [30].

miR-224 is a microRNA commonly dysregulated in most human cancers that affects crucial cellular processes and resides in chromosome Xq28 [22]. The intriguing role of miR-224 has yet to be fully elucidated. It may depend on the expression of miR-224 target genes in various cell types, since it can promote or inhibit cancer cell growth, depending on the histotype of the malignancy concerned. Some surveys recently suggested that miR-224 overexpression could play a significant part in promoting tumor cell proliferation and migration, with an oncogenic role. Yu et al. found miR-224 upregulation and AKT activation synergistically associated with tumor progression in hepatocellular carcinoma (HCC) [25]. Lan et al. found that HCV-induced low autophagy could lead to a high miR-224 expression with a tumorigenic effect [16]. Huang et al. suggested that miR-224 might have an important role in promoting the onset and progression of bone metastases from breast cancer [31]. miR-224 is an independent prognostic marker in cervical cancer and lung cancer too [17, 19, 32]. On the other hand, Mencia et al. demonstrated that miR-224 underexpression leads to insensitivity towards methotrexate, favoring a resistant phenotype [18]. In glioblastoma, miR-224 overexpression increases radiation sensitivity, thus improving outcomes, and patients with high miR-224 expression levels reportedly have a better overall survival [20]. Finally, miR22-4 downregulation was found to promote tumor progression in prostate cancer [33]. It is also worth mentioning the recent finding that, although miR-224 upregulation is a known negative prognostic factor in HCC, the high miR-224 phenotype in this cancer has been found associated with a better response to sorafenib, an inhibitor of several tyrosine kinase receptors, such as RET, RAF kinase, and vascular endothelial growth factor (VEGF) receptor, that is also used in MTC [34]. In the light of the above findings, and as demonstrated for other miRNAs, such as miR-221 and miR-222 [35-37], miR-224 seems to have a dual mode of action that depends on the type of cancer involved: it acts as an onco-miR in some cancers and as an oncosuppressor-miR in others, suggesting that different molecular targets and networks are regulated by miR-224 in different neoplastic scenarios. As Croce put it, “before describing a miRNA as a tumor suppressor or an oncogene, it is necessary to specify in which cell or tissue, as cellular context is crucial for the function of miRNAs” [38].

To our knowledge, this is the first study to explore the expression profile of miR-224 in a large cohort of MTC patients, assessing its relationship with clinical, pathological, and molecular features. The literature currently provides little or no information on miR-224 expression levels in neuroendocrine tumors. A previous study of ours on a small series of 34 MTC patients found that miR-224 could represent a prognostic biomarker associated with a better outcome [11]. Our present findings confirmed as much, since miR-224 expression levels were significantly lower in patients with distant metastases, persistent disease, and disease progression during their follow-up. More importantly, as already shown in glioblastoma tissues, we ascertained that miR-224 expression levels was significantly associated with patient survival. Our Kaplan-Meier analysis showed that patients with tumors showing a low miR-224 expression levels were likely to have a significantly shorter overall survival than those with a high miR-224 expression levels, strongly suggesting that low miR-224 expression levels is a marker of a poor prognosis in patients with MTC. In association with serum Ct levels at diagnosis and stage of disease, miR-224 emerged from our multivariate analysis as an independent prognostic marker.

Our findings also support the positive prognostic role of *RAS* mutation found in a previous multicenter study [8]: all of our *RAS*-mutated patients were alive, and they had a less aggressive MTC phenotype and higher levels of miR-224 expression (although, based on our data, we cannot rule out the possibility of such associations being accidental). Finally, in line with other reports, we found that *RET* and *RAS* mutations were mutually exclusive [39–42].

*RET* activation stimulates multiple downstream pathways, promoting cell growth, proliferation, survival, and differentiation [43]. Two of the main pathways involved are the mitogen-activated protein kinase (MAPK) and the phosphoinositide 3-kinase (PI3K)/AKT pathways [43]. It remains to be seen whether *RAS* mutations can also lead to the activation of both of these signaling pathways in sporadic MTC, or whether there is a preferential activation pathway. Lyra et al. examined mTOR activation in a series of 87 MTCs (10 familial and 77 sporadic) and found *RAS* mutations significantly associated with a more intense expression of phospho-S6 ribosomal protein (p-S6, a downstream effector of mTOR) [44], pointing to an association between mTOR pathway activation and the presence of *RAS* mutations in MTC. We consequently conducted a recent study and demonstrated that MTC-harboring *RAS* mutations showed a preferential activation of the PI3K/Akt/mTOR pathway, revealed by an intense phospho-Akt reactivity pattern on Western blot analysis [24]. The different outcomes in *RAS*- versus *RET*-mutated patients could be due to these different activation pathways. The present study showed that, together with the presence of RAS mutations, higher miR-224 expression levels are also molecular markers of a favorable prognosis in sporadic MTC. Further studies are now needed to clarify whether these two factors are both implicated in the same signaling network.

In the clinical setting, Ct-doubling time and CEA-doubling time represent the most important markers for predicting the behavior of MTC [4], but these parameters change over time, being longer when the disease is in its early stages and shorter in later stages, when there is disease progression [8]. The molecular characterization of sporadic MTC at diagnosis, based on a search for somatic *HRAS*, *KRAS*, *NRAS*, or *RET* mutations, or miRNA expression profiling, will hopefully pave the way to a customized patient follow-up from the outset in the near future [3, 8]. In addition, the discovery of miRNAs offers a novel mechanism for different treatment options and efforts to develop new modulators capable of inhibiting oncogenic miRNAs by using miRNA antagonists (anti-miRs), or by introducing a tumor suppressor miRNA mimetic to restore a loss of function [45–47]. Judging from our data, miR-224 acts as an oncosuppressor-miR in MTC and lower miR-224 expression levels serves as an independent prognostic molecular marker of a more aggressive disease that can identify patients at risk of progression and MTC-related death. Thanks to its stability and the reliability with which it can be assayed in different types of specimen (plasma, cytology, and frozen tissue), miR-224 could represent an ideal marker in MTC, enabling an improvement in patients' risk stratification from the start.

## Figures and Tables

**Table 1:** Clinical, pathological, and molecular data on MTC cases.

	MTC
Total number	134
Gender	
Male	54/134 (37%)
Female	80/134 (43%)
Median age (95%CI)	58.5 (55–61)
Median follow-up in months (95%CI)	40 (32–42)
Median calcitonin level in pg/ml at diagnosis (95%CI)	466 (5.5–22931)
Stage	
I	62/134 (46%)
II	26/134 (19%)
III	13/134 (10%)
IV	33/134 (25%)
Lymph node involvement	
Present	46/134 (34%)
Absent	88/134 (66%)
Distant metastases	
Present	12/134 (9%)
Absent	122/134 (91%)
Germline RET mutation	
Present	27/134 (20%)
Absent	107/134 (80%)
Sporadic RET mutation	
Present	39/107 (36%)
Absent	68/107 (64%)
Sporadic RAS mutation	
Present	10/107 (9%)
Absent	94/107 (91%)
Biochemical cure^1^	
Present	86/133 (65%)
Absent	35/133 (35%)
Disease progression	
Present	17/133 (13%)
Absent	116/133 (87%)
Disease-related death^2^	9/132 (7%)

^1^Ct level was unavailable for one patient. ^2^Two patients were lost to follow-up.

**Table 2:** Clinical data based on RET and RAS mutational status in sporadic MTCs.

	RET+/RAS− *n* = 39 (%)	RET−/RAS− *n* = 58 (%)	RAS+/RET− *n* = 9^1^ (%)	*p*
Median calcitonin level in pg/ml at diagnosis (*n* = 106)	700 (83–22931)	331 (20–42300)	402 (61–747)	0.01
Stage			
I-II (*n* = 66)	18 (46%)	41 (71%)	7 (78%)	0.03
III-IV (*n* = 40)	21 (54%)	17 (29%)	2 (22%)	
Lymph node metastases (*n* = 40)	21 (54%)	17 (29%)	2 (22%)	0.03
Biochemical cure^2^ (*n* = 64)	18 (46%)	38 (67%)	8 (89%)	0.03
Disease progression (*n* = 15)	9 (24%)	6 (10%)	0	0.08
Disease-related death^3^ (*n* = 9)	7 (18%)	2 (4%)	0	0.03

^1^One patient with a novel RAS mutation was excluded from statistical analysis. ^2^Ct level was unavailable for one patient. ^3^Two patients were lost to follow-up.
